# Defining Appropriate Resources for Nursing Homes to Reduce Potentially Avoidable Transfers After a Fall

**DOI:** 10.1093/geroni/igab046.2125

**Published:** 2021-12-17

**Authors:** Raphaëlle Guerbaai, Michael Simon, Franziska Zúñiga

**Affiliations:** 1 Institute of Nursing Science, Department of Public Health, University of Basel, Institute of Nursing Science, Department of Public Health, University of Basel, Basel-Stadt, Switzerland; 2 University of Basel, Basel, Basel-Stadt, Switzerland

## Abstract

Falls are common in nursing home (NH) residents and are the predominant reason for an emergency department (ED) transfer. Falls are responsible for 25% - 87% of ED transfers, a proportion of which are potentially avoidable. INTERCARE – an implementation science study reducing unplanned hospitalizations (2018 – 2020) – involved experts to identify potentially avoidable fall-related transfers. Focus group and stakeholder survey enabled identification of resources to safely manage some falls in NHs. 25.9% of fall-related transfers were potentially avoidable based on using root-cause analysis and discharge reports. Avoidability was associated to ED visit, compared to hospitalizations. Appropriate resources identified by stakeholders included timely access to outpatient services for diagnostic imaging (e.g., X-Ray) and clinical skills’ training in suturing and wound care for registered or specialist nurses. Although NHs are striving for a home-like environment, better access to basic diagnostic and treatment services within NHs should be possible.

